# Impact of COVID-19 Preventative Measures on Otolaryngology in Taiwan: A Nationwide Study

**DOI:** 10.3390/ijerph20043371

**Published:** 2023-02-14

**Authors:** Hsiao-Yun Cho, Chia-Hung Hung, Yi-Wei Kao, Ben-Chang Shia, Mingchih Chen

**Affiliations:** 1 Department of Otorhinolaryngology-Head and Neck Surgery, Fu Jen Catholic University Hospital, Fu Jen Catholic University, No. 69, Guizi Road, Taishan Distict, New Taipei City 24352, Taiwan; 2 Graduate Institute of Business Administration, College of Management, Fu Jen Catholic University, No. 510, Zhongzhen Road, Xinzhuang District, New Taipei City 24205, Taiwan; 3 School of Medicine, College of Medicine, Fu Jen Catholic University, New Taipei 24205, Taiwan; 4 Department of Orthopedics, Fu Jen Catholic University Hospital, Fu Jen Catholic University, New Taipei City 24352, Taiwan; 5 Artificial Intelligence Development Center, Fu Jen Catholic University, No. 510, Zhongzheng Road, Xinzhuang District, New Taipei City 24205, Taiwan; 6 Department of Applied Statistics and Information Science, Ming Chuan University, Taoyuan City 32462, Taiwan

**Keywords:** COVID-19, Taiwan, otolaryngology

## Abstract

Background: Taiwan always had low case rates of COVID-19 compared with other countries due to its immediate control and preventive measures. However, the effects of its policies that started on 2020 for otolaryngology patients were unknown; therefore, the aim of this study was to analyze the nationwide database to know the impact of COVID-19 preventative measures on the diseases and cases of otolaryngology in 2020. Method: A case-compared, retrospective, cohort database study using the nationwide database was collected from 2018 to 2020. All of the information from outpatients and unexpected inpatients with diagnoses, odds ratios, and correlation matrix was analyzed. Results: The number of outpatients decreased in 2020 compared to in 2018 and 2019. Thyroid disease and lacrimal system disorder increased in 2020 compared to 2019. There was no difference in carcinoma in situ, malignant neoplasm, cranial nerve disease, trauma, fracture, and burn/corrosion/frostbite within three years. There was a highly positive correlation between upper and lower airway infections. Conclusions: COVID-19 preventative measures can change the numbers of otolaryngology cases and the distributions of the disease. Efficient redistribution of medical resources should be developed to ensure a more equitable response for the future.

## 1. Introduction

In December 2019, an unknown virus broke out in Wuhan, China, which spread globally at a rapid speed [1,2,3]. The World Health Organization (WHO) named this virus COVID-19, which is caused by severe acute respiratory syndrome coronavirus 2 (SARS-CoV-2) that is mainly transmitted through respiratory droplets and contact routes between people [1,4]. With Taiwan being geographically in close proximity to China and having a densely urbanized population, it was predicted to be one of the hardest-hit countries. However, as Taiwan immediately took decisive and effective control measures to prevent the spread from the very start of the pandemic, the people of Taiwan are now able to continue their normal lives, with precaution [2,3]. At the end of 2020, the Taiwan Centers for Disease Control (CDC) confirmed that, of a total of 799 cases, 704 were imported cases and only 7 people died [5].

Taiwan continues to take control measures for combating the pandemic; the fundamental prevention measures include: prompt border control, social distancing, postponement of large crowd events, and compulsory wearing of surgical masks when outside [2,6,7]. The key principles of the “Taiwan model” are rapid measures, early deployment, prudent actions, and transparency [8,9]. From several previous studies, epidemic prevention strategies can effectively reduce severe respiratory infectious diseases, such as invasive pneumonia disease, tuberculosis, and influenza [10,11]. It also significantly reduced dengue and enterovirus infections [10,12]; however, there were no statistical decreases in the human immunodeficiency virus (HIV) and hepatitis C virus (HCV) infections.

As the pandemic continues, specialists and related staff that deal with significantly increased numbers of hospital patients are at high risk for COVID-19 infection. For specialists that work specifically in close contact with airway management, such as otolaryngology, their examination and treatments are unavoidable for patients. In addition, this has greatly impacted the declining numbers of outpatient visits, delivery of patient care, reduction in waiting time, decrease in identifying new cancer, and significant delays in treatment deliveries [13,14,15]. As COVID-19 hit hard in 2020 for Taiwan, and currently, there is no literature discussing the effects of inpatients and outpatients, or surgeries in otolaryngology with COVID-19 occurrence that had not yet become an epidemic with under surveilled prevention policies. This is the first nationwide study with the aim to disclose the impact of COVID-19 preventative measures on the diseases of otolaryngology. Our hypothesis is that COVID-19 preventative measures can change the numbers of otolaryngology cases and the distributions of the diseases. This study will not only explore the effects of Taiwan’s epidemic prevention measures, but will also add value to future structural hospital procedures, clinical evidence-based decision, and healthcare policy making in knowing how to plan the allocation of medical resources during future possible pandemics.

## 2. Materials and Methods

### 2.1. Data Source and Ethical Consideration

Taiwan’s National Health Insurance Research Database (NHIRD) was used in this study. This database, which was established in 1995, represents most of the Taiwanese population, if not all, as it contains the data of approximately 23 million residents (over 99%). The NHIRD has often been used by hundreds of published studies [16], providing abundant information related to all aspects of healthcare, from ambulatory and inpatient care to outpatient care and medication data, creating real-world evidence to support healthcare policy-making and clinical decisions. The International Classification of Diseases 9 Revision Clinical Modification (ICD-9-CM) was used for all the diagnosis codes, and the 10th revision of ICD-10-CM started in 2016. According to the ethical guidelines from the NHIRD, all individual patient material was anonymized before accessing it; therefore, the requirements for informed consent were waived by the Research Ethics Committee. The Institutional Review Board and Ethics Committee of Fu Jen Catholic University was approved (IRB approval number: No. C110199).

### 2.2. Study Population and Disease Grouping

**Figure 1:**
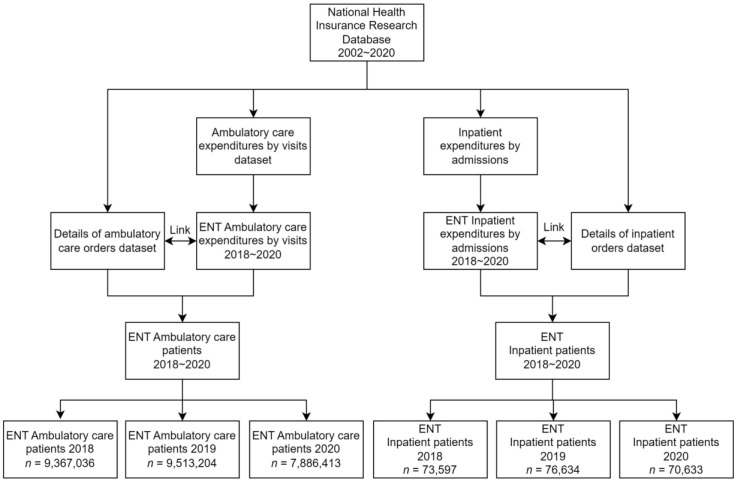
Research flow diagram of the database from the National Health Insurance Research Database.

This study was a case-compared, retrospective, cohort database study to detrmine the before and after effects of COVID-19 prevention measures on infectious and non-infectious diseases. The database search included otolaryngology inpatients and outpatient data from 2018 to 2020, collecting all the different diagnoses and categories of surgical procedures, and the number of visits, as seen in Figure 1. The inpatient and outpatient operations are included according to the operation codes in the National Health Insurance Payment Standard. The total inpatient and outpatient cases are identified in Table 1 and Table 2. All operations that were performed by otolaryngologists from 2018 to 2020 were included in this study, and were then divided into inpatient groups and outpatient groups, according to whether the patients were hospitalized. All groupings of the diagnoses of the outpatients in the otolaryngology department are in Appendix A, Table A1. An additional analysis of unexpected inpatient diagnoses in the otolaryngology department was performed. All patient characteristics were available from the database.

### 2.3. Statistics

All statistical analyses were analyzed using the R programming software (R software, version 4.0.3, Vienna, Austria). Descriptive statistics were used to evaluate the counts and percentages of the outcome variables. The Chi-square test was used to test the relevance of age, gender, region, and salary level. An odds ratio was used to calculate the change in the ratio of inpatient and outpatient diagnoses each year in order to calculate the correlation strength. The year 2019 was used as the benchmark for analysis. The Pearson correlation coefficient was used to calculate the correlation between each diagnosis. Additional growth rate and trend check Poisson regression calculations were analyzed and are shown in Table A3 and Table A4 of Appendix A. The growth rates were formulated by subtracting the initial month and the end month and then dividing that by the initial month (for example, where January is the initial month and February is the end month). The unit of growth rate is a percentage. Poisson regression was used when the trend direction was consistent Therefore, the *p*-value was blank if it was not analyzed. The difference was considered significant if the two-sided *p*-value was ≤0.05.

## 3. Results

Table 1 and Table 2 display the baseline characteristics of gender, age group, area, and monthly salary from 2018 to 2020 for inpatient and outpatient otolaryngology cases. There were more outpatient cases than inpatient cases throughout all three years, with 2018 having 9,367,036 compared to 73,597, 2019 having 9,513,204 compared to 76,634, and 2020 having 7,886,413 outpatient cases compared to 70,633 inpatient cases. There were significant differences in age group, area, and monthly salary for both inpatient and outpatient otolaryngology cases. Only outpatient otolaryngology cases displayed additional significant differences for gender.

Table 3 and Table 4 show the total numbers of outpatients and unexpected inpatients along with their odds ratios from 2018 to 2020. Thyroid disease and lacrimal system disorder persistently increased from 2018 to 2020 in the outpatient diagnoses groups. The groups that had no significant changes from 2019 to 2020 were malignant neoplasm, trauma and fracture, cranial nerve disease, carcinoma in situ, and burn/corrosion/frostbite. The groups that had a significant decrease from 2019 to 2020 were lower and upper airway infection, voice and speech disorder, chronic lower airway disorder, otitis media, acute gastroenteritis, and chronic rhino sinusitis. There were no unexpected inpatient diagnosis groups that had persistent increases from 2018 to 2020.

**Figure 2:**
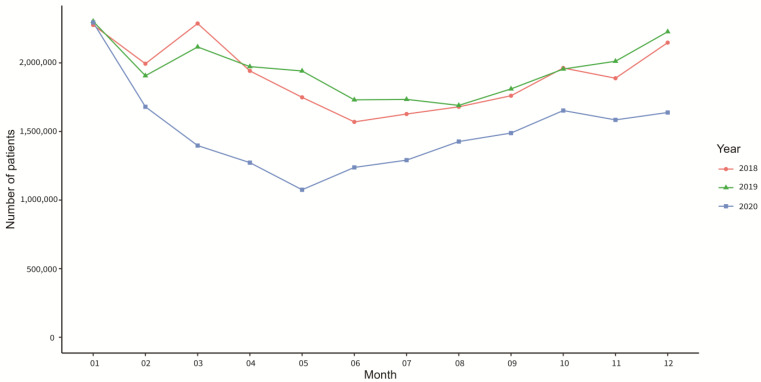
Total number of otolaryngology outpatients in each month from 2018 to 2020.

Figure 2 displays the monthly number of outpatients from 2018 to 2020. There was a major decreasing number of cases from January 2020 to May 2020, until it slowly increased from June 2020 to December 2020.

**Figure 3:**
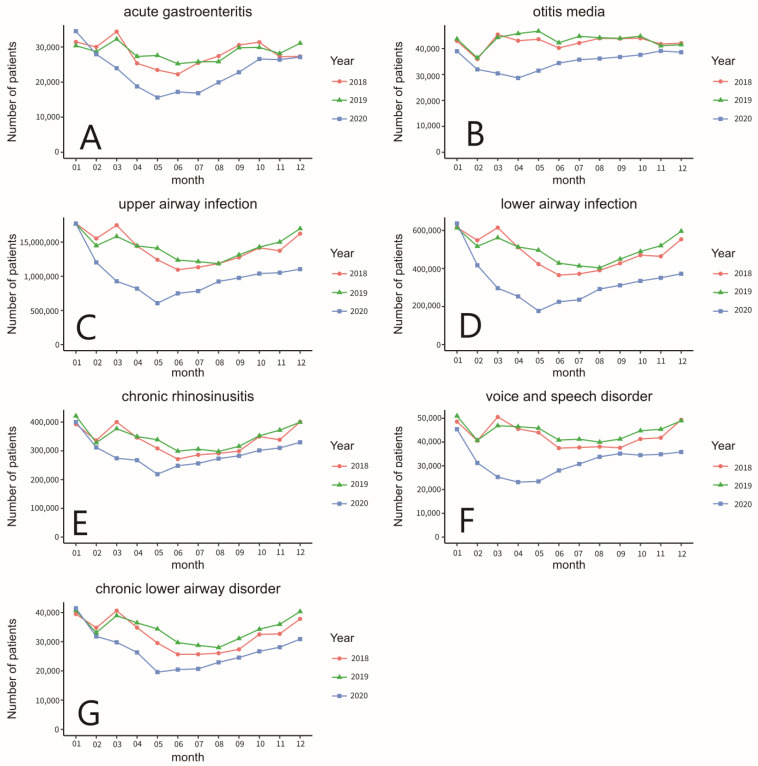
(**A**) The total number of outpatients of acute gastroenteritis, (**B**) otitis media, (**C**) upper airway infection, (**D**) lower airway infection, (**E**) chronic rhinosinusitis, (**F**) voice and speech disorder, and (**G**) chronic lower airway disorder from 2018 to 2020.

**Figure 4:**
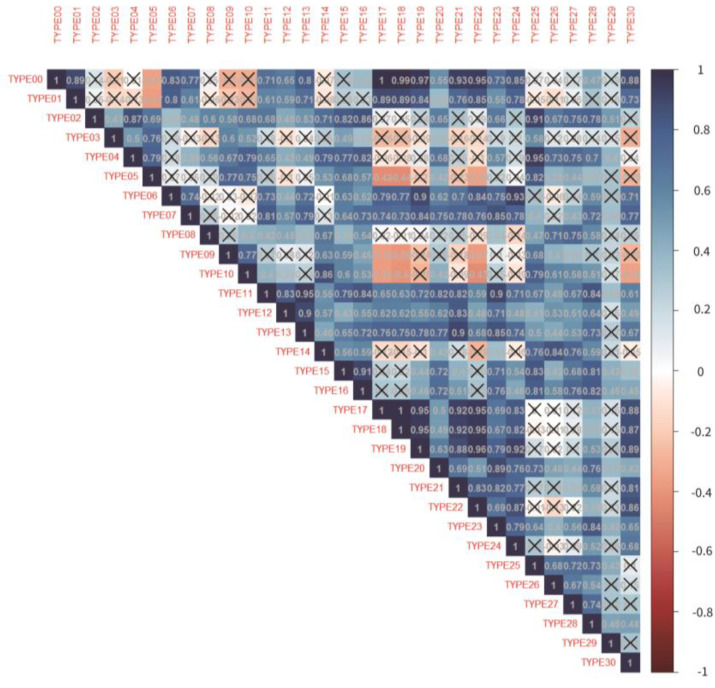
Correlation matrix between the diagnoses grouping. (“X” in the correlation matrix represents *p* > 0.05).

Figure 3 shows the monthly number of patients from the top seven outpatient diagnoses groups that had a decrease from 2019 to 2020. Growth rate and trend check of each monthly comparison throughout all three years of the outpatient diagnoses group are shown in Table A3 in Appendix A. Figure 4 shows the correlation matrix between each outpatient diagnosis group. The type code numbers are according to the diagnosis grouping in Appendix A, Table A2.

**Figure 5:**
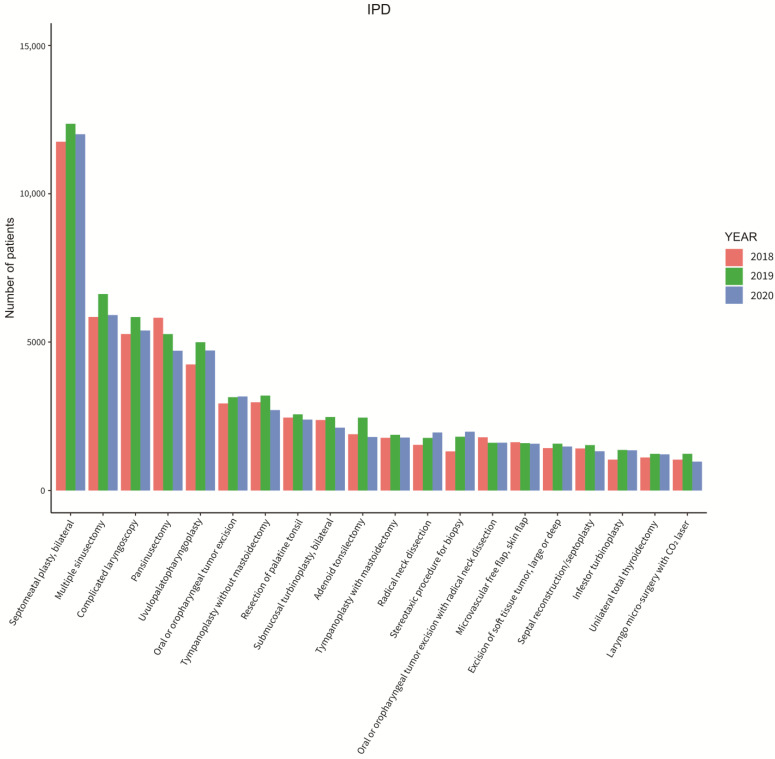
Total otolaryngology surgeries performed on inpatients in 2018 to 2020.

**Figure 6:**
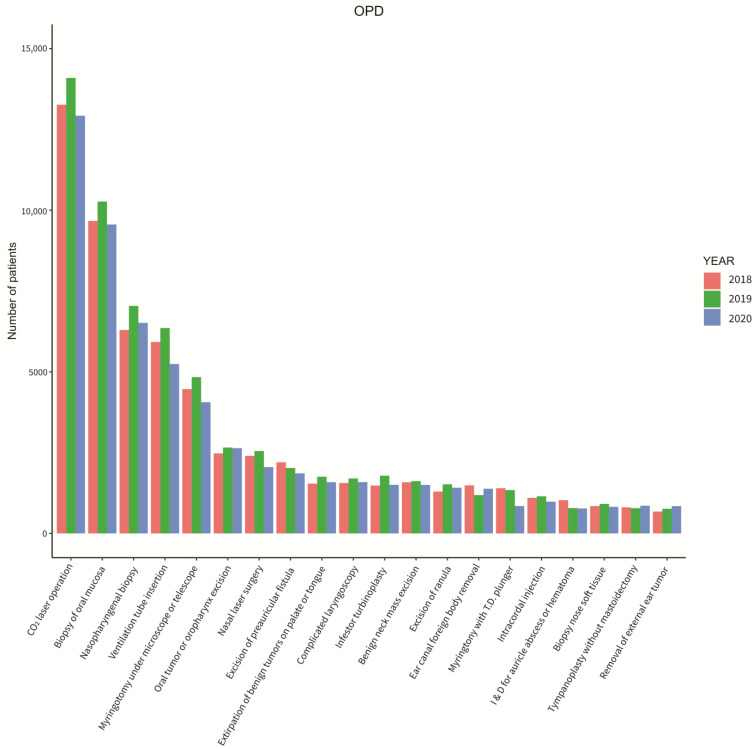
Total otolaryngology surgeries performed on outpatients in 2018 to 2020.

**Figure 7:**
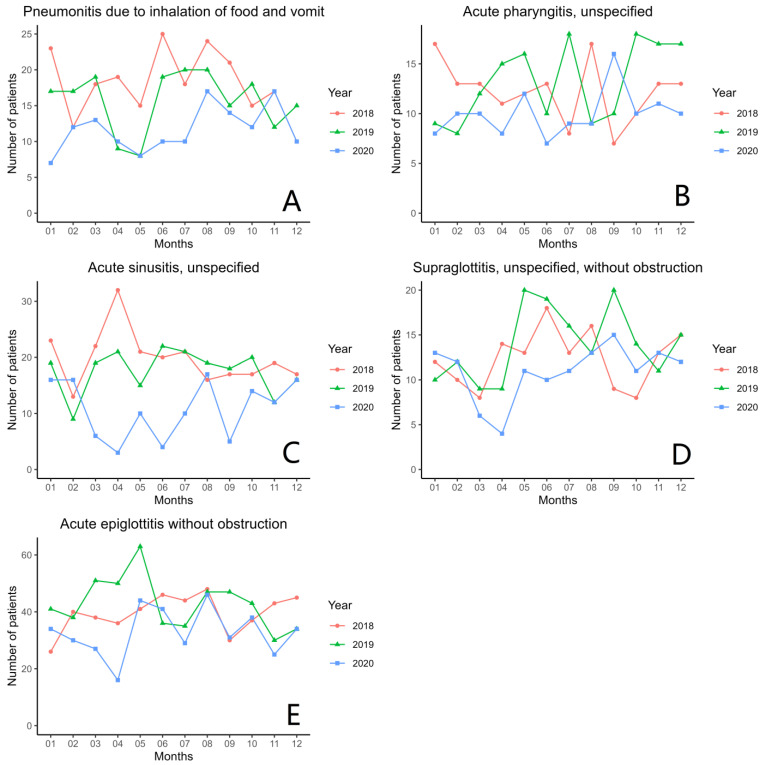
The total number of unexpected inpatient diagnoses of (**A**) pneumonitis, (**B**) acute pharyngitis, (**C**) acute sinusitis, (**D**) supraglottitis, and (**E**) acute epiglottitis from 2018 to 2020.

Figure 5 and Figure 6 show the different types of surgeries that were operated on from 2018 to 2020 by inpatients and outpatients. The most common surgeries that were performed were bilateral septomeatal plasty in inpatient cases and CO_2_ laser operation in outpatient cases throughout all three years. Coming in second was the biopsy of oral mucosa for outpatients throughout all three years. Lastly, Figure 7 displays the six most common unexpected inpatient diagnoses throughout each month from 2018 to 2020. An additional growth rate and trend check of each monthly comparison throughout all three years of the unexpected inpatient diagnoses are shown in Table A4 of Appendix A.

## 4. Discussion

It is unclear what the effect on Taiwan would have been if it had undergone a significant COVID-19 outbreak with under-surveilled prevention policies, as Taiwan had immediate control measures for combating the pandemic. As there was no current literature discussing this issue in the last few years, to our knowledge, this study is the first to use the national real-world database to analyze and disclose the impact of COVID-19 and Taiwan’s epidemic prevention policy on otolaryngology patients and surgeries.

In this study, the number of outpatient visits in 2018 and 2019 is relatively similar in each month. From December to March, the number of visits is higher, and from June to August, it is lower overall. The most common diseases that are seen in otolaryngology clinics are upper airway infection, lower airway infection, and chronic rhinosinusitis, according to Table 3. The peak season for these diseases is usually in the winter, where there is a significant decrease in temperature, while in the summer from June to August, there is a significant increase in temperature and a decrease in patient visits. However, in 2020, the numbers from this study are different than in 2018 and 2019. Since the outbreak of COVID-19 in January 2020, Taiwan immediately implemented a mask-wearing regulation in February 2020, vigorously advocated social distancing, and executed stringent border control [6]. Due to these measures, the number of patient visits to the otolaryngology clinics significantly decreased from February until May, there was a slow increase back to typical levels from 2018 and 2019, as shown in Figure 2. Possibly, unnecessary fear and panic may have been present at the beginning of the pandemic among patients seeking medical treatment. In addition, the government’s and CDC’s recommendation to not go to the hospital unless necessary could be another factor for the decrease in numbers. From June to October 2020, the numbers returned to similar levels as two years ago, but the overall number is still significantly smaller. In December, the peak of hospital visits and the number of patients can be seen to decrease significantly. This could be explained by the effective epidemic prevention advocated by the government and the CDC, which has not only reduced the rapid spread of COVID-19, but also greatly reduced the common respiratory diseases seen in hospital visits.

Previous literature has discussed that the willingness of outpatients to see a doctor greatly decreased due to COVID-19. In Table 3, most of the diagnosis groups can be seen to decrease from 2019 to 2020; however, some diagnoses groups did not show a change in continuing to seek medical treatment. Groups such as thyroid disease, malignant neoplasm of the head and neck, in situ carcinoma, cranial nerve disease, and burn/corrosion/frostbite of the head, face, and neck require long-term follow-up with long-term drug prescriptions. At that time, patients may have maintained regular hospital visits and would not hesitate to seek medical treatment, due to possible serious effects if help was not sought.

From Figure 2, this study saw many significant reductions in outpatient visits in 2020. There were obvious differences in lower airway infection, upper airway infection, voice and speech disorder, chronic lower airway disorder, otitis media, acute gastroenteritis, and chronic rhino sinusitis. By examining further each month from 2018 to 2020, in Figure 3, it can be found that upper and lower airway infection and voice and speech disorders, which originally had seasonal differences, showed a gradual, but noticeable, increase in the second half of 2020, in comparison with chronic rhino sinusitis and lower airway disorder. While the overall outpatient visits were declining, the seasonal increase remains similar in 2020 to that of 2018 and 2019. This observation shows that the Taiwan epidemic prevention policy that came effective immediately had a strong influence on acute respiratory infection symptoms, but also, significantly reduced chronic upper and lower airway disorders.

Additional correlation statistics between all outpatient diagnoses groupings were analyzed and it was found the upper and lower airway infections had a completely positive correlation. This indicated that these two types of diseases tend to occur in the same season with the same transmission route, showing the same change trend under the epidemic situation. Similar discussions were also examined from a 2018 clinical study, examining the correlation between upper and lower airway inflammations, suggesting that the inflammatory process may be the most essential link in the cross-talk between the upper and lower airways [17]. There were also positive correlations between upper and lower airway infections, chronic rhino sinusitis, voice and speech disorders, acute gastroenteritis, and chronic lower airway disorders. This indicated that these diseases were more likely to appear together simultaneously and have a tendency to increase and decrease at the same time. Lastly, the unusual correlation was the negative correlation between thyroid disease, lacrimal system disorders, and otitis externa with chronic lower airway disorders, upper airway infections, and lower airway infections. This may be explained by the possible fear of patients not wanting to go to the hospitals for respiratory-related treatments during the epidemic. However, for diseases such as thyroid, lacrimal system disorders, and otitis externa, which are less likely to cause droplet infections, there are still related medical needs, and therefore, there may be a tendency to diverge. 

Figure 5 and Figure 6 did not indicate additional information from the number of patients according to the different surgical procedures that were performed. Throughout all three years, there were no differences, which may indicate that under the prevention measure controls, Taiwan’s medical resources are still sufficient with the public to continue to be confident in the medical treatment provided.

From 2019 to 2020, the top five unexpected inpatient diagnoses that were evaluated remained the same, including abscess and cellulitis of the face and neck, sialoadenitis, vertigo, sudden hearing loss, and Bell’s palsy. These diagnoses did not affect the patients who needed to be hospitalized to receive active treatment, nor will they reduce the incidence of the disease.

Since the development of the COVID-19 pandemic, the increase of previous studies has clearly shown that COVID-19 itself and many related drugs and vaccines have some side effects, many of which are related to otolaryngology [18,19]. Common otolaryngology symptoms of COVID-19 patients may include tinnitus, gingivitis, sudden hearing loss, Bell’s palsy, and hoarseness [20]. In addition, the side effects of drug and COVID-19 vaccine usage include systemic event reactions, injection site adverse reactions, and serious vaccine-related adverse events. Their data also show to have symptoms such as dizziness, hearing loss, vertigo, tinnitus, cough, rhinorrhea, throat irritation, pharyngalgia, nasal congestion, blurry vision with dizziness, and dysgeusia [21,22,23]. However, this study is mainly focused on Taiwan’s population, which has not yet experienced a pandemic in 2020, when the total number of confirmed cases was only 799 [5]. Relevant drugs and vaccines have not come out nor are widely used in Taiwan, so the related side effects had no effect, as shown by the results of this study.

This study had the advantage of using a national population dataset algorithm that included a large-scale sample size. This study allows people to understand the impact of the COVID-19 pandemic on Taiwan’s epidemic prevention policy on otolaryngology outpatients and unexpected inpatient hospitalizations. By analyzing all the diseases and surgeries that occurred from 2018 to 2020, this study also evaluated the further changes in different months with a complete correlation of the outpatient diagnoses. 

The limitations for this study were, firstly, that it did not include an experimental comparison. It was unclear whether the epidemic panic from the patients or the epidemic prevention measures can have a significant impact on the related diseases. Future design studies are worth investigating in providing stronger evidence of the post-epidemic era so that government and healthcare policymakers can have more references for formulating regulations and allocating medical resources. Secondly, because Taiwan did not declare a pandemic in 2020, and as telemedicine was not commonly used, it was impossible to know whether the preventive measures will also have an impact on these patients and the types of diseases. Lastly, this study focused on the analysis of otolaryngology patients. If possible, future studies should analyze whether the preventative measures in Taiwan have the same impact on other departments.

## 5. Conclusions

COVID-19 preventative measures can change the numbers of otolaryngology cases and the distributions of the diseases. Therefore, if a global pandemic will happen again in the future, we hope that complete effective measures and policies for epidemic prevention can maintain the maintain the medical capacity and reduce common infectious respiratory diseases. At the same time, medical institutions should put more medical resources and time into providing regular follow-up, long-term drug treatments, and cancer management, as well as routinely supply the treatment of emergency traumatic illnesses, to ensure a more equitable response for the future.

## Figures and Tables

**Table 1:** Baseline characteristics of inpatient otolaryngology cases in 2018 to 2020.

Variable	Categories	2018 (*n*)	2019 (*n*)	2020 (*n*)	*p*	Missing
Total Cases		73,597	76,634	70,633		
Gender	Female	26,654 (37.0%)	27,903 (37.2%)	25,609 (37.1%)	0.862	2.1
	Male	45,333 (63.0%)	47,192 (62.8%)	43,499 (62.9%)		
Age Group	0–18	4657 (6.3%)	4992 (6.5%)	3646 (5.2%)	<0.001	0.0
	19–64	56,891 (77.3%)	58,660 (76.5%)	54,523 (77.2%)		
	65+	12,049 (16.4%)	12,982 (16.9%)	12,464 (17.6%)		
Area	North	33,099 (47.6%)	33,938 (47.1%)	30,882 (46.2%)	<0.001	5.7
	Central	18,716 (26.9%)	20,206 (28.0%)	18,668 (28.0%)		
	South	15,806 (22.8%)	16,042 (22.3%)	15,412 (23.1%)		
	East	1670 (2.4%)	1674 (2.3%)	1584 (2.4%)		
	Other	185 (0.3%)	209 (0.3%)	237 (0.4%)		
Salary (NTD)	0	323 (0.4%)	472 (0.6%)	284 (0.4%)	<0.001	0.0
	1–15,840	15,302 (20.8%)	15,598 (20.4%)	14,502 (20.5%)		
	15,840–25,000	28,127 (38.2%)	28,187 (36.8%)	25,610 (36.3%)		
	>25,000	29,845 (40.6%)	32,377 (42.2%)	30,237 (42.8%)		

Abbreviation: NTD: New Taiwan Dollars.

**Table 2:** Baseline characteristics of outpatient otolaryngology cases in 2018 to 2020.

	Categories	2018 (*n*)	2019 (*n*)	2020 (*n*)	*p*	Missing
Total Cases		9,367,036	9,513,204	7,886,413		
Gender	Female	4,906,173 (53.2%)	4,972,284 (53.1%)	4,118,768 (53.1%)	<0.001	1.6
	Male	4,317,163 (46.8%)	4,390,035 (46.9%)	3,636,311 (46.9%)		
Age Group	0–18	1,816,322 (19.4%)	1,790,043 (18.8%)	1,485,620 (18.8%)	<0.001	0.0
	19–64	6,380,756 (68.1%)	6,484,905 (68.2%)	5,282,535 (67.0%)		
	65+	1,169,958 (12.5%)	1,238,256 (13.0%)	1,118,258 (14.2%)		
Area	North	4,594,882 (52.5%)	4,683,105 (52.8%)	3,882,939 (52.6%)	<0.001	6.6
	Central	1,947,923 (22.3%)	1,968,899 (22.2%)	1,641,972 (22.2%)		
	South	160,205 (1.8%)	160,975 (1.8%)	137,763 (1.9%)		
	East	2,022,765 (23.1%)	2,037,347 (23.0%)	1,706,731 (23.1%)		
	Other	19,694 (0.2%)	20,306 (0.2%)	16,837 (0.2%)		
Salary (NTD)	0	55,295 (0.6%)	75,864 (0.8%)	43,404 (0.6%)	<0.001	0.0
	1–15,840	1,613,030 (17.2%)	1,636,515 (17.2%)	1,367,257 (17.3%)		
	15,840–25,000	3,487,570 (37.2%)	3,371,047 (35.4%)	2,716,655 (34.4%)		
	>25,000	4,211,141 (45.0%)	4,429,778 (46.6%)	3,759,097 (47.7%)		

Abbreviation: NTD: New Taiwan Dollars.

**Table 3:** Total numbers of outpatients and odds ratios in each diagnosis group from 2018 to 2020.

Diagnoses Group	2018 (*n*)	2019 (*n*)	2020 (*n*)	Odds Ratio in 2018 (95% CI)	Odds Ratio in 2020 (95% CI)
*Increase persistently from 2019 to 2020*					
Thyroid disease	31,473	35,059	38,295	0.900 (0.887, 0.914) *	1.094 (1.078, 1.110) *
Lacrimal system disorder	1078	1140	1239	0.949 (0.873, 1.031)	1.088 (1.004, 1.180) *
*No difference from 2019 to 2020*					
Malignant neoplasm of head and neck	56,359	57,279	56,869	0.987 (0.975, 0.998) *	0.994 (0.983, 1.006)
Trauma and fracture of head, face and neck	17,585	16,782	16,841	1.051 (1.029, 1.074) *	1.005 (0.984, 1.027)
Cranial nerve disease	6797	6192	6272	1.101 (1.064, 1.140) *	1.014 (0.979, 1.051)
Carcinoma in situ	184	229	240	0.806 (0.663, 0.978) *	1.049 (0.876, 1.258)
Burn/corrosion/frostbite of head, face and neck	123	116	123	1.063 (0.825, 1.372)	1.062 (0.824, 1.370)
*Decreasing from 2019 to 2020*					
Lower airway infection	3,196,002	3,316,407	2,253,312	0.961 (0.960, 0.963) *	0.647 (0.646, 0.648) *
Upper airway infection	7,607,692	7,739,052	5,856,342	0.979 (0.978, 0.981) *	0.680 (0.679, 0.680) *
Voice and speech disorder	369,189	381,885	263,493	0.969 (0.965, 0.974) *	0.688 (0.684, 0.691) *
Chronic lower airway disorder	227,607	242,497	183,088	0.941 (0.935, 0.946) *	0.754 (0.750, 0.759) *
Otitis media	322,413	329,513	261,347	0.981 (0.976, 0.986) *	0.792 (0.788, 0.796) *
Acute gastroenteritis	263,525	269,775	220,240	0.980 (0.974, 0.985) *	0.816 (0.811, 0.820) *
Chronic rhino sinusitis	2,247,645	2,320,289	1,931,434	0.969 (0.967, 0.971) *	0.819 (0.817, 0.821) *
Eustachian tube disorder	215,779	222,360	186,289	0.973 (0.967, 0.979) *	0.838 (0.833, 0.843) *
Gastroesophageal inflammation and ulcer	798,147	883,271	754,008	0.903 (0.900, 0.906) *	0.850 (0.847, 0.853) *
Sleep disorder	191,897	195,815	171,260	0.983 (0.977, 0.989) *	0.875 (0.869, 0.881) *
Other unspecified disease	456,211	438,243	384,449	1.045 (1.041, 1.049) *	0.876 (0.873, 0.880) *
Chronic laryngopharyngeal disorder	91,298	101,291	88,812	0.904 (0.896, 0.912) *	0.878 (0.870, 0.886) *
Headache	314,265	330,363	298,417	0.954 (0.949, 0.958) *	0.903 (0.899, 0.908) *
Other disease of external ear	829,479	848,497	767,562	0.980 (0.977, 0.983) *	0.903 (0.900, 0.906) *
Oral cavity and salivary gland disorder	589,787	609,723	551,857	0.969 (0.966, 0.973) *	0.904 (0.901, 0.908) *
Congenital deformity	7237	7048	6691	1.030 (0.997, 1.064)	0.951 (0.919, 0.983) *
Foreign body of head and neck	217,335	217,557	206,815	1.002 (0.996, 1.008)	0.952 (0.946, 0.957) *
Other disease of middle ear	25,757	25,924	24,673	0.997 (0.980, 1.014)	0.953 (0.937, 0.970) *
Hearing loss and tinnitus	398,731	409,350	391,463	0.977 (0.972, 0.981) *	0.957 (0.953, 0.961) *
Benign neoplasm	32,168	33,604	32,147	0.960 (0.946, 0.975) *	0.958 (0.943, 0.973) *
Vertigo	516,618	537,690	523,948	0.963 (0.959, 0.967) *	0.975 (0.972, 0.979) *
Skin/subcutaneous tissue/lymphatic disorder	278,644	289,382	282,235	0.965 (0.960, 0.970) *	0.976 (0.971, 0.981) *
Otitis externa	651,451	677,400	674,152	0.964 (0.960, 0.967) *	0.996 (0.993, 0.999) *

Odds Ratio in 2019 is used as 1.000 reference.* Odd Ratio *p* < 0.05.

**Table 4:** Total numbers of unexpected inpatient diagnoses and odds ratios in each diagnosis group from 2018 to 2020.

Unexpected Inpatient Diagnoses	2018 (*n*)	2019 (*n*)	2020 (*n*)	Odds Ratio in 2018 (95% CI)	Odds Ratio in 2020 (95% CI)
*Decreasing numbers from 2019 to 2020*					
Acute sinusitis, unspecified	233	203	125	1.151 (0.954, 1.390)	0.617 (0.493, 0.770) *
Pneumonitis due to inhalation of food and vomit	199	179	130	1.115 (0.911, 1.365)	0.728 (0.579, 0.911) *
Acute pharyngitis, unspecified	143	155	116	0.925 (0.737, 1.162)	0.750 (0.588, 0.953) *
Acute epiglottitis without obstruction	465	510	388	0.915 (0.806, 1.037)	0.762 (0.667, 0.869) *
Supraglottitis, unspecified, without obstruction	149	165	127	0.906 (0.725, 1.130)	0.771 (0.611, 0.971) *
Local infection of the skin and subcutaneous tissue, unspecified	193	197	156	0.983 (0.805, 1.199)	0.793 (0.642, 0.978) *
Acute tonsillitis, unspecified	1876	1787	1611	1.053 (0.987, 1.123)	0.903 (0.844, 0.966) *
Sudden idiopathic hearing loss	2164	2047	1860	1.060 (0.998, 1.126)	0.910 (0.854, 0.969) *
*No difference from 2019 to 2020*					
Aspergillosis, unspecified	65	93	100	0.702 (0.509, 0.961) *	1.077 (0.812, 1.430)
Unspecified mycosis	670	710	698	0.947 (0.852, 1.052)	0.984 (0.887, 1.093)
Klebsiella pneumoniae [K. pneumoniae] as the cause of diseases classified elsewhere	83	78	97	1.067 (0.783, 1.456)	1.245 (0.925, 1.681)
Bell’s palsy	189	168	157	1.128 (0.917, 1.390)	0.936 (0.752, 1.163)
Acute recurrent tonsillitis, unspecified	89	123	126	0.726 (0.551, 0.953) *	1.026 (0.800, 1.316)
Pneumonia, unspecified organism	405	382	354	1.063 (0.925, 1.223)	0.928 (0.803, 1.072)
Abscess, furuncle and carbuncle of nose	91	95	75	0.961 (0.720, 1.282)	0.791 (0.583, 1.070)
Peritonsillar abscess	1464	1376	1371	1.067 (0.991, 1.149)	0.998 (0.926, 1.075)
Edema of larynx	137	128	134	1.073 (0.843, 1.367)	1.048 (0.823, 1.337)
Retropharyngeal and parapharyngeal abscess	381	364	317	1.050 (0.909, 1.212)	0.872 (0.750, 1.014)
Other abscess of pharynx	164	140	129	1.175 (0.938, 1.474)	0.923 (0.726, 1.172)
Other diseases of pharynx	775	895	927	0.869 (0.789, 0.956) *	1.037 (0.946, 1.137)
Acute respiratory failure, unspecified whether with hypoxia or hypercapnia	115	119	95	0.969 (0.750, 1.253)	0.800 (0.609, 1.047)
Sialoadenitis, unspecified	459	447	418	1.030 (0.904, 1.173)	0.936 (0.819, 1.070)
Acute sialoadenitis	134	129	107	1.042 (0.818, 1.328)	0.831 (0.642, 1.073)
Abscess of salivary gland	187	171	167	1.097 (0.891, 1.350)	0.978 (0.790, 1.211)
Cellulitis and abscess of mouth	665	691	655	0.965 (0.868, 1.074)	0.949 (0.853, 1.056)
Cutaneous abscess of face	237	210	210	1.132 (0.940, 1.364)	1.001 (0.827, 1.213)
Cutaneous abscess of neck	332	334	314	0.997 (0.856, 1.161)	0.941 (0.807, 1.098)
Cellulitis of face	695	701	730	0.994 (0.895, 1.104)	1.043 (0.940, 1.157)
Cellulitis of neck	489	454	437	1.080 (0.951, 1.228)	0.964 (0.845, 1.099)
Acute lymphadenitis of face, head and neck	170	154	140	1.107 (0.890, 1.378)	0.910 (0.724, 1.145)
Inflammatory conditions of jaws	238	268	257	0.891 (0.748, 1.061)	0.960 (0.809, 1.140)
Epistaxis	417	372	382	1.124 (0.978, 1.293)	1.028 (0.891, 1.186)
Dyspnea, unspecified	191	276	254	0.694 (0.577, 0.834) *	0.922 (0.777, 1.093)
Dizziness and giddiness	552	491	468	1.128 (0.999, 1.274)	0.955 (0.841, 1.083)

Odds ratio in 2019 is used as 1.000 reference.* Odd Ratio *p* < 0.05.

## Data Availability

These data were available to us as staffs of the Department of Otolaryngology at Fu Jen Catholic University Hospital and at Fu Jen Catholic University, using the National Hospital Research Database (NHIRD). These data are protected by the Ministry of Health and Welfare and patient privacy laws in Taiwan. No public links of the NHIRD are available; however, they will be made available after the appropriate data privacy and human subject approvals needed by the institution. Requests and questions should be sent to 081438@mail.fju.edu.tw.

## Appendix A

**Table A1:** ICD-10-CM codes of diagnoses of the outpatients in the otolaryngology department.

Diagnosis	ICD Code
1. Acute Gastroenteritis	ICD-10-CM: A04, A05, A08, A09
2. Malignant neoplasm of head and neck	ICD-10-CM: C00-14, C30-32, C43, C49.0, C72.2, C72.4, C76.0, C81-86
3. Carcinoma in situ	ICD-10-CM: D00, D02, D03
4. Benign neoplasm	ICD-10-CM: D10-11, D14, D17-18, D21, D23, D37-38
5. Thyroid disease	ICD-10-CM: C73, D34, D44.0, E04-06
6. Headache	ICD-10-CM: G43-44, R51
7. Sleep disorder	ICD-10-CM: F51, G47
8. Cranial nerve disease	ICD-10-CM: G50-52
9. Lacrimal system disorder	ICD-10-CM: H04
10. Otitis externa	ICD-10-CM: H60.0-60.3, H60.5-60.6, H60.8-60.9
11. Other disease of external ear	ICD-10-CM: H60.4, H61.0-61.3, H61.8-61.9
12. Otitis media	ICD-10-CM: H65.0-65.4, H66, H92, H70
13. Eustachian tube disorder	ICD-10-CM: H68-69
14. Other disease of middle ear	ICD-10-CM: H71-74, H80
15. Vertigo	ICD-10-CM: H81-83, R42
16. Hearing loss and tinnitus	ICD-10-CM: H90-91, H93
17. Upper airway infection	ICD-10-CM: B27, B30.2, J00-06, J09-11, J36, J39, R05-07, R50
18. Lower airway infection	ICD-10-CM: J12-16, J18, J20-22
19. Chronic rhino sinusitis	ICD-10-CM: J30-34, R09.81-09.82
20. Chronic laryngopharyngeal disorder	ICD-10-CM: J35, J37, R13
21. Voice and speech disorder	ICD-10-CM: F80, J38, R47, R49
22. Chronic lower airway disorder	ICD-10-CM: J40, J42-45, J47
23. Oral cavity and salivary gland disorder	ICD-10-CM: B37.0, B37.83, K05, K09, K11-14
24. Gastroesophageal inflammation and ulcer	ICD-10-CM: K20-21, K25-29
25. Skin/subcutaneous tissue/lymphatic disorder	ICD-10-CM: B00, B02, B07, L02-04, L20, L72, R22, R59
26. Congenital deformity	ICD-10-CM: Q16-18, Q30-32, Q35-38
27. Trauma and fracture of head, face and neck	ICD-10-CM: S00.3-00.5, S00.8-00.9, S01.2-01.5, S01.8-01.9, S02-03, S04.5-04.6, S08.1, S08.8, S09.2-09.3, S09.9, S10, S11.01, S11.03, S11.1-11.2, S11.8-11.9, S17, S19
28. Foreign body of head and neck	ICD-10-CM: T16, T17.0-17.3, T18.0
29. Burn/corrosion/frostbite of head, face and neck	ICD-10-CM: T20, T27, T28.0, T28.41, T28.5, T28.91, T33.0-33.1, T34.0-34.1, T49.6
30. Other unspecified disease	ICD-10-CM: M26.6, M35.0, R04, R43, Z01.1, Z96.2

**Table A2:** Diseases according to the type code.

Type Code	Diseases
TYPE01	Acute gastroenteritis
TYPE02	Malignant neoplasm of head and neck
TYPE03	Carcinoma in situ
TYPE04	Benign neoplasm
TYPE05	Thyroid disease
TYPE06	Headache
TYPE07	Sleep disorder
TYPE08	Cranial nerve disease
TYPE09	Lacrimal system disorder
TYPE10	Otitis externa
TYPE11	Other disease of external ear
TYPE12	Otitis media
TYPE13	Eustachian tube disorder
TYPE14	Other disease of middle ear
TYPE15	Vertigo
TYPE16	Hearing loss and tinnitus
TYPE17	Upper airway infection
TYPE18	Lower airway infection
TYPE19	Chronic rhinosinusitis
TYPE20	Headache
TYPE21	Voice and speech disorder
TYPE22	Chronic lower airway disorder
TYPE23	Oral cavity and salivary gland disorder
TYPE24	Gastroesophageal inflammation and ulcer
TYPE25	Skin/subcutaneous tissue/lymphatic disorder
TYPE26	Congenital deformity
TYPE27	Trauma and fracture of head, face and neck
TYPE28	Foreign body of head and neck
TYPE29	Burn/corrosion/frostbite of head, face and neck
TYPE30	Other unspecified disease

**Table A3:** Outpatient diagnoses monthly growth rate comparison.

Outpatient Diagnoses Grouping/ Monthly Comparison	2018 Growth Rate (%)	2019 Growth Rate (%)	2020 Growth Rate (%)	2018 vs. 2019	2019 vs. 2020
*Acute gastroenteritis*					
January to February	−4.5	−5.6	−19.0	<0.001	<0.001
February to March	14.6	12.4	−14.2	<0.001	
March to April	−26.2	−15.3	−21.6	<0.001	<0.001
April to May	−7.5	1.0	−16.9		
May to June	−5.2	−8.5	10.3	<0.001	
June to July	14.6	2.2	−2.2	<0.001	
July to August	7.6	0.1	18.3	<0.001	<0.001
August to September	11.5	15.4	14.5	<0.001	0.01
September to October	2.6	0.3	16.6	<0.001	<0.001
October to November	−13.1	−5.8	−0.7	<0.001	<0.001
November to December	0.3	10.4	2.8	<0.001	<0.001
*Otitis media*					
January to February	−16.3	−16.7	−18.0	0.163	<0.001
February to March	26.4	21.6	−4.8	<0.001	
March to April	−5.3	3.4	−5.8		
April to May	1.3	2.0	9.8	<0.001	<0.001
May to June	−7.7	−9.6	9.3	<0.001	
June to July	4.7	5.9	3.8	<0.001	<0.001
July to August	4.3	−1.2	1.2		
August to September	−0.3	−0.5	1.7	<0.001	
September to October	0.4	1.9	2.2	<0.001	<0.001
October to November	−5.1	−8.2	4.0	<0.001	
November to December	0.8	1.0	−1.3	<0.001	
*Upper airway infection*					
January to February	−12.4	−18.0	−32.0	<0.001	<0.001
February to March	12.5	9.3	−22.9	<0.001	
March to April	−17.4	−8.7	−11.6	<0.001	<0.001
April to May	−13.9	−2.3	−26.1	<0.001	<0.001
May to June	−11.8	−12.3	23.7	<0.001	
June to July	3.3	−1.8	4.6		
July to August	4.7	−2.4	17.9		
August to September	7.6	10.8	5.7	<0.001	<0.001
September to October	11.0	8.7	6.4	<0.001	<0.001
October to November	−3.0	5.2	1.3		<0.001
November to December	18.2	13.1	4.9	<0.001	<0.001
*Lower airway infection*					
January to February	−11.0	−15.9	−34.5	<0.001	<0.001
February to March	12.2	8.8	−28.9	<0.001	
March to April	−16.9	−8.6	−14.6	<0.001	<0.001
April to May	−17.2	−3.4	−30.2	<0.001	<0.001
May to June	−13.7	−13.7	27.2	0.728	
June to July	1.9	−3.4	4.9		
July to August	5.0	−2.4	23.8		
August to September	9.4	11.4	6.7	<0.001	<0.001
September to October	10.1	8.8	7.4	<0.001	<0.001
October to November	−1.3	6.3	4.8		<0.001
November to December	19.3	14.7	6.1	<0.001	<0.001
*Chronic rhinosinusitis*					
January to February	−14.3	−22.1	−22.0	<0.001	0.474
February to March	19.0	14.9	−12.1	<0.001	
March to April	−13.5	−7.3	−2.5	<0.001	<0.001
April to May	−10.9	−3.1	−18.2	<0.001	<0.001
May to June	−12.1	−11.8	13.4	<0.001	
June to July	5.5	2.3	3.3	<0.001	<0.001
July to August	1.8	−2.7	6.5		
August to September	2.7	6.0	3.5	<0.001	<0.001
September to October	16.9	11.7	6.7	<0.001	<0.001
October to November	−3.2	5.5	2.8		<0.001
November to December	18.4	7.5	6.3	<0.001	<0.001
*Voice and speech disorder*					
January to February	−16.5	−20.2	−31.2	<0.001	<0.001
February to March	24.5	15.1	−19.0	<0.001	
March to April	−9.9	−0.8	−8.5	<0.001	<0.001
April to May	−3.4	−1.3	1.3	<0.001	
May to June	−14.9	−11.0	19.6	<0.001	
June to July	0.8	0.9	9.7	0.012	<0.001
July to August	0.9	−3.0	9.8		
August to September	−1.2	3.2	4.0		<0.001
September to October	9.9	8.5	−1.9	<0.001	
October to November	1.2	1.4	1.1	0.023	<0.001
November to December	18.1	7.9	2.7	<0.001	<0.001
*Chronic lower airway disorder*					
January to February	−11.9	−18.7	−23.2	<0.001	<0.001
February to March	16.8	17.7	−6.4	0.008	
March to April	−14.3	−6.3	−11.6	<0.001	<0.001
April to May	−15.2	−5.7	−25.6	<0.001	<0.001
May to June	−13.1	−13.6	4.4	0.072	
June to July	0.1	−3.2	1.2		
July to August	1.4	−2.6	10.8		
August to September	5.0	11.1	7.1	<0.001	<0.001
September to October	18.7	10.3	8.7	<0.001	<0.001
October to November	0.6	4.9	5.3	<0.001	0.028
November to December	15.7	12.2	9.9	<0.001	<0.001

**Table A4:** Unexpected inpatient diagnoses monthly growth rate comparison.

Unexpected Inpatient Diagnoses/ Monthly Comparison	2018 Growth Rate (%)	2019 Growth Rate (%)	2020 Growth Rate (%)	2018 vs. 2019	2019 vs. 2020
*Pneumonitis due to inhalation of food and vomit*					
January to February	−47.8	0.0	71.4		
February to March	50.0	11.8	8.3	0.054	0.777
March to April	5.6	−52.6	−23.1		0.198
April to May	−21.1	−11.1	−20.0	0.561	0.626
May to June	66.7	137.5	25.0	0.09	0.013
June to July	−28.0	5.3	0.0		
July to August	33.3	0.0	70.0		
August to September	−12.5	−25.0	−17.6	0.333	0.632
September to October	−28.6	20.0	−14.3		
October to November	13.3	−33.3	41.7		
November to December	−41.2	25.0	−41.2		
*Acute pharyngitis, unspecified*					
January to February	−23.5	−11.1	25.0	0.492	
February to March	0.0	50.0	0.0		
March to April	−15.4	25.0	−20.0		
April to May	9.1	6.7	50.0	0.826	0.034
May to June	8.3	−37.5	−41.7		0.862
June to July	−38.5	80.0	28.6		0.174
July to August	112.5	−50.0	0.0		
August to September	−58.8	11.1	77.8		0.034
September to October	42.9	80.0	−37.5	0.349	
October to November	30.0	−5.6	10.0		
November to December	0.0	0.0	−9.1		
*Acute sinusitis, unspecified*					
January to February	−43.5	−52.6	0.0	0.669	
February to March	69.2	111.1	−62.5	0.299	
March to April	45.5	10.5	−50.0	0.039	
April to May	−34.4	−28.6	233.3	0.715	
May to June	−4.8	46.7	−60.0		
June to July	5.0	−4.5	150.0		
July to August	−23.8	−9.5	70.0	0.257	
August to September	6.3	−5.3	−70.6		0.001
September to October	0.0	11.1	180.0		<0.001
October to November	11.8	−40.0	−14.3		0.174
November to December	−10.5	33.3	33.3		1
*Supraglottitis, unspecified, without obstruction*					
January to February	−16.7	20.0	−7.7		
February to March	−20.0	−25.0	−50.0	0.806	0.317
March to April	75.0	0.0	−33.3	0.009	0.083
April to May	−7.1	122.2	175.0		0.455
May to June	38.5	−5.0	−9.1		0.668
June to July	−27.8	−15.8	10.0	0.433	
July to August	23.1	−18.8	18.2		
August to September	−43.8	53.8	15.4		0.096
September to October	−11.1	−30.0	−26.7	0.338	0.855
October to November	62.5	−21.4	18.2		
November to December	15.4	36.4	−7.7	0.306	
*Acute epiglottitis without obstruction*					
January to February	53.8	−7.3	−11.8		0.53
February to March	−5.0	34.2	−10.0		
March to April	−5.3	−2.0	−40.7	0.401	<0.001
April to May	13.9	26.0	175.0	0.226	<0.001
May to June	12.2	−42.9	−6.8		<0.001
June to July	−4.3	−2.8	−29.3	0.712	0.005
July to August	9.1	34.3	58.6	0.013	0.15
August to September	−37.5	0.0	−32.6		
September to October	23.3	−8.5	22.6		
October to November	16.2	−30.2	−34.2		0.752
November to December	4.7	13.3	36.0	0.203	0.085
